# Cognitive Decline in Parkinsonism: From Clinical Phenotypes to the Genetic Background

**DOI:** 10.3390/biomedicines13071624

**Published:** 2025-07-02

**Authors:** Christos Koros, Evangelia Stanitsa, Efthalia Angelopoulou, Sokratis G. Papageorgiou, Leonidas Stefanis

**Affiliations:** 1st Department of Neurology, Eginition Hospital, National and Kapodistrian University of Athens, 11528 Athens, Greece; eva.st.92@gmail.com (E.S.); angelthal@med.uoa.gr (E.A.); sokpapa@med.uoa.gr (S.G.P.); lstefanis@bioacademy.gr (L.S.)

**Keywords:** genetic, cognitive decline, dementia, Parkinson’s disease, dementia with Lewy bodies, atypical parkinsonian syndromes, frontotemporal dementia

## Abstract

**Background/Objectives**: Cognitive impairment often occurs in various parkinsonian syndromes. The course of deficits in cognitive functions ranges from mild cognitive decline to severe deterioration. Affected cognitive domains are also variable. The genetic background of patients exhibiting parkinsonism with concomitant cognitive decline is still elusive. A significant part of current research in Parkinson’s disease and other parkinsonian syndromes is targeted towards the genetic aspects of these disorders. The aim of the present review was to summarize existing studies focusing on the investigation of the interplay between genetic data in parkinsonism and associated cognitive symptoms. **Methods**: A review of English-language articles published between 2000 and 2024 was conducted, focusing on genetic studies of Parkinson’s disease and atypical parkinsonian syndromes with cognitive decline, using the databases PUBMED, SCOPUS, and EMBASE. **Results**: We have selected a clinical phenotype-wise assessment of parkinsonian conditions with cognitive deficits, including typical or early-onset Parkinson’s disease, dementia with Lewy bodies, Corticobasal Syndrome, Progressive Supranuclear Palsy, and frontotemporal dementia with parkinsonism. Both typical and atypical parkinsonian syndromes with concomitant cognitive decline were explored. **Conclusions**: Genetic background likely contributes to the heterogeneity of cognitive impairment in parkinsonian syndromes, with specific mutations linked to distinct cognitive symptoms. The integration of genetic data and a more thorough neuropsychological assessment with clinical, imaging, and biomarkers may enhance diagnosis and enable personalized therapies.

## 1. Introduction

Cognitive deterioration is a prevalent feature in both idiopathic and genetic forms of Parkinson’s disease (PD), as well as atypical parkinsonian syndromes. Cognitive decline often occurs early in PD, with approximately 20–33% of patients exhibiting mild cognitive impairment (MCI) at the time of diagnosis, and more than 50% progressing to dementia during the course of the disease [1]. Key factors associated with cognitive decline in PD are advanced age and prolonged disease duration. Among the cognitive domains that are usually impaired are attention and visuospatial functions, with variable impact on memory which reflects significant heterogeneity in cognitive performance among patients [2,3].

Genetic factors play a crucial role in this cognitive variability, alongside comorbidities [2,3]. In addition to documented pathogenic mutations in PD genes, common genetic variants in at least three genes, apolipoprotein E (*APOE*), microtubule-associated protein tau (*MAPT*), and α-synuclein (*SNCA*), may play a role in the eventual susceptibility to cognitive impairment in PD patients. Furthermore, histopathological studies highlight cortical and limbic deposition of pathological α-synuclein as central for PD dementia. However, co-existing pathologies such as beta-amyloid plaques, tau protein-related pathology, and vascular lesions may contribute to the cognitive decline in PD. Despite that most cases of PD are sporadic, up to 10–15% of patients have a family history, suggesting a significant genetic contribution. The genetic basis of PD is complex and includes monogenic forms of PD and genetic risk factors [4]. Autosomal dominant PD is associated with mutations in the gene for α-synuclein (*SNCA*), leucine-rich repeat kinase 2 (*LRRK2*), and vesicular sorting protein 35 (*VPS35*). Common genes causing autosomal recessive PD include *PRKN*, PTEN-induced kinase 1 (*PINK1*), and *DJ-1*. Genetic risk factors for PD have also been identified, with pathogenic mutations in the glucocerebrosidase (*GBA*) gene representing the main one.

In related disorders, such as dementia with Lewy bodies (DLB), genetic factors like *GBA* and *APOE* are recognized as determinants in the pathogenesis. Moreover, in atypical parkinsonian syndromes, like Progressive Supranuclear Palsy (PSP) or Corticobasal Syndrome (CBS), cognitive deterioration leading to dementia may be one of the main aspects of the disease’s phenotype. Genetic forms of these syndromes have also been reported, most notably for CBS, although these cases are frequently sporadic. Finally, clear-cut dementia disorders like genetic forms of frontotemporal dementia (FTD) may manifest parkinsonian motor components.

The aim of the present review was to consolidate the existing studies focusing on the investigation of the interplay between genetic data in parkinsonism and associated cognitive decline. This review adopts a clinical phenotype-wise approach to genetic parkinsonian conditions ranging from typical or early-onset Parkinson’s disease with variable cognitive deficits to atypical parkinsonism including dementia with Lewy bodies (DLB), Corticobasal Syndrome (CBS), and Progressive Supranuclear Palsy (PSP), and genetic forms of frontotemporal dementia (FTD) with parkinsonism.

## 2. Materials and Methods

We reviewed articles published during the 2000–2024 period, focusing on genetic data in patients with PD and atypical parkinsonian syndromes exhibiting cognitive decline. We searched PUBMED, SCOPUS, and EMBASE from 2000 to 2024 using the following terms: “Genetic”, “Parkinson’s disease”, “Atypical parkinsonian syndrome”, “Dementia with Lewy Bodies”, “Corticobasal degeneration”, “Frontotemporal dementia with parkinsonism”, “Glucocerebrosidase gene”, “Alpha-Synuclein gene”, “ Leucine-rich repeat kinase 2”, “*VPS35*”, “Parkin”, “*PRKN*”, “*PINK1*”, “*DJ-1*”, “*APOE*”. Our literature search focused on studies assessing cognitive functions in carriers of pathogenic mutations manifesting PD or atypical parkinsonism. Only articles published in English have been included in the present review.

## 3. Results

### 3.1. Genetic Forms of PD

#### 3.1.1. Typical Mid-/Late-Onset Genetic PD with Variable Cognitive Decline

##### *LRRK2* Mutation Carriers

Mutations in the *LRRK2* gene are the most common cause of autosomal dominant PD. In certain populations a higher prevalence has been reported, for instance approximately 20% in the case of patients of Ashkenazi Jewish descent. Pathogenic mutations include p.G2019S, p.R1441C/G/H, and others [5,6]. *LRRK2*-PD is clinically similar to idiopathic PD (henceforth iPD), although some differences have been reported, such as fewer nonmotor symptoms (sleep, autonomic), favorable response to L-DOPA, a relatively late age at onset, and lack of atypical signs. Previous detailed studies, which included an extensive neuropsychological assessment, have shown variable results regarding cognitive decline in *LRRK2* carriers [2,3]. However, some studies have not shown any marked differences in cognitive deterioration between *LRRK2*-PD and iPD. According to a large study of Ashkenazi Jews both with and without the p.G2019S *LRRK2* mutation, there were not any significant differences in general cognitive ability based on the administration of the Montreal Cognitive Assessment (MoCA) test [7]. In contrast, other studies have shown superior cognitive performance among patients with *LRRK2*-PD as compared to iPD [2,3].

Alcalay and co-authors reported higher scores of *LRRK2*-PD p.G2019S carriers compared to iPD patients on tests assessing executive functions and language [8]. In another study p.G2019S and p.R144G *LRRK2* PD carriers performed better than iPD patients in working memory [9]. Furthermore, *LRRK2* PD patients carrying the p.R1441G mutation exhibited higher scores than iPD patients on tests of episodic verbal memory [10]. The neuropathological profile in *LRRK2*-PD possibly accounts for the relatively preserved cognitive functions in *LRRK2* carriers compared to iPD patients, given the fact that a proportion of *LRRK2* patients do not have Lewy body pathology. Notably, previous studies support the idea that the presence of LB pathology is related to cognitive impairment [8]. However, in *LRRK2* carriers the heterogeneity of results might derive from different mutations of the studied population, different demographic characteristics (age, sex) of patients participating in various studies, and the variety of the cognitive tests performed. The aforementioned data support a trend towards milder cognitive decline in *LRRK2* carriers compared to iPD patients [7].

##### *VPS35* Mutation Carriers

Next-generation sequencing allowed the identification of a heterozygous mutation in the *VPS35* gene, p.Asp620Asn, associated with the development of PD. This mutation is found in only 0.4% of PD patients [11]. In terms of phenotype, patients with *VPS35* are similar to those with sporadic PD, with the exception of earlier onset at 50–60 years of age [11,12]. *VPS35* encodes protein sorting-related protein 35 which, together with other proteins, forms the retromer complex involved in vesicular recycling. It has various roles within the neuron in pathways corresponding to the handling of α-synuclein and is also important for mitochondrial function [13].

There are only a few studies investigating the cognitive functions in patients with *VPS35* mutations possibly due to the rarity of these genetic abnormalities in PD [2,3]. To date, case series investigating the cognitive and neuropsychiatric features of *VPS35* have shown that MCI might occur in the majority of patients with the mutation, while there were patients that had neurotypical performance in cognitive tests [12,14].

##### *APOE* 

The association between the E4 allele of the APOE apolipoprotein gene and Alzheimer’s disease is well established. In recent years, studies using extensive neuropsychological tests have shown faster cognitive decline with *APOE* e4 carriers in patients with PD [15,16]. *APOE* e4 was recently shown to act as a genetic modifier and increase cognitive burden in patients with iPD and in SNCA mutation carriers, while this was not the case for the *GBA* mutation carriers. Patients with the E4 allele were more likely to develop dementia [17,18]. Genetic analysis with a genome-wide association study (GWAS) has also played a role in investigating the relationship of *APOE* E4, dementia, and PD [19,20,21]. Based on the above, the *APOE* e4 genotype is associated with cognitive dysfunction in PD, and further studies are needed to improve our understanding of the underlying mechanism.

#### 3.1.2. Early- or Mid-Onset Genetic PD with Variable Cognitive Decline

##### *SNCA* Mutation Carriers

Mutations in the *SNCA* gene were the first genetic cause linked to familial PD. However, the frequency of *SNCA* mutations as a genetic cause is very low. Several point mutations (including p.A53T, p.A53E, p.A53V, p.A30P, p.A30G, p.E46K, p.H50Q, and p.G51D) have been described as well as duplications and triplications of the gene related to PD.

The clinical phenotype is variable according to the mutation. The majority of *SNCA*-PD patients described cognitive impairment and dementia with psychiatric symptoms such as visual hallucinations or occasional delusions. Systematic reviews have shown that the prevalence of dementia varies according to the type of *SNCA* mutation, at approximately 40% in p.A53T and as high as 88–100% in cases of triplication in the *SNCA* gene [3]. However, these percentages should be interpreted with caution; evaluations are not based on homogenous criteria and cases are few. What is more, disease duration, which appears to be the main predictor of cognitive decline in these cases [24], varies across studies. Overall, PD in patients carrying *SNCA* mutations is associated with an earlier age of disease onset, faster motor progression, earlier onset of motor fluctuations, and occurrence of nonmotor symptoms compared to iPD [4].

Most of the relevant information derives from the study of carriers of the p.A53T *SNCA* mutation. The disease, if it occurs in such carriers, generally has a typical course, but some variability still remains [22,23]. The average age of onset is 46 years. The disease has a penetrance of approximately 80–90% [24]. Overall, carriers of the mutation have a similar but more aggressive course than in the idiopathic form (iPD). The cognitive phenotype is usually consistent with PD, PD with dementia, or DLB. Cognitive decline is variable, but dementia usually occurs within 5–7 years of disease onset [24,25,26,27,28]. A case with a DLB phenotype has been described with this mutation [29]. In a recently published longitudinal 3-year analysis of 16 p.A53T-PD and 48 iPD cases from the Parkinson’s Progression Markers Initiative (PPMI), baseline data for global cognitive function, as assessed by the MoCA, were not significantly different between the two groups, in contrast to tests evaluating executive/visuospatial function, which were worse in A53T-PD. These results are consistent with an earlier study [30]. Longitudinally, there was a significant decline in all neuropsychological tests in A53T-PD, while in iPD these remained relatively stable [31]. This reinforces the notion that after a period of 4–6 years in which A53T-PD quite closely resembles typical iPD, cognitive decline is usually accelerated thereafter, despite some notable variability [24]. In terms of additional genetic factors that could play a role in such variability, interestingly, more severe cognitive decline in A53T-PD is associated with the *APOE*-E4 allele [19].

Although the pattern of dementia in p.A53T PD is compatible with dementia in PD, some cases show atypical features: A Swedish patient developed, within a few years from the disease onset, severe language dysfunction, resembling Primary Progressive Aphasia (PPA) [32]. Also, a study by our clinic has reported an atypical pattern of initial disease manifestation with an FTD-like picture with behavioral and speech dysfunction in two p.A53T *SNCA* carriers, expanding the phenotypic spectrum [33].

Other SNCA point mutations are very rare. The recently reported p.A30G mutation in the Greek population manifests with a rather typical PD phenotype, including levodopa-responsive parkinsonism, age of onset varying from 36 to 80 years old, with a mean age around 55 years of age, and various prominent nonmotor symptoms, such as psychiatric manifestations, orthostatic hypotension, and REM sleep behavior disorder. The presence of dementia is variable, but most cases exhibit some degree of cognitive decline as assessed by global cognitive tests like the MMSE and the MoCA [34,35]. The p.E46K mutation has been reported in a family of Spanish Basque origin [36]. Affected patients show a rather severe phenotype with high penetrance. Dementia with a DLB phenotype and autonomic dysregulation is characteristic. Symptoms begin between the ages of 50 and 65, and dementia occurs within a few years, although there is considerable variability. Neuropsychological assessment has shown that posterior cortical dysfunction may be a distinct early feature of cognitive impairment [37]. In another family, motor and nonmotor manifestations were significantly more benign [38]. In carriers of the p.A30P mutation, found in a German family, the clinical phenotype was like that of iPD with onset around 60 years and a more benign course. Nonmotor symptoms were not reported except for cognitive impairment, which was present in half of affected patients [39]. In addition, neuropsychological tests have provided evidence of cognitive decline as a frequent and early symptom [40]. The p.H50Q SNCA mutation has been reported in families of English descent. In a family, patients exhibited rather typical motor manifestations of PD at age 60 years and mild cognitive decline along with apathy 4 years later, while some had a more rapid course of dementia within 5 years [41]. Another p.H50Q late-onset case developed dementia 9 years after disease onset [42]. Another recently described mutation, p.G51D, has been described in several families with different ethnic backgrounds [12,43,44] and may have a variable atypical presentation and course, including very early age of onset at age 19 in one case, and pyramidal tract findings, myoclonus, and seizures. Psychiatric symptoms, dementia, and autonomic dysfunction are common, but not always present. A novel p.V15A variant has been identified in one Turkish, one Italian, and two Japanese families, characterized by relatively early age of onset, and a parkinsonian phenotype accompanied by cognitive decline and visual hallucinations [45,46].

In addition to point mutations, multiplications of the *SNCA* gene are also responsible for hereditary PD in families in which several members were affected. Severity of clinical symptoms correlates with *SNCA* copy numbers, with triplication cases showing more deficits and earlier disease onset. *SNCA* triplication results in severe PD with concomitant cognitive impairment and was first described in families in Iowa [47,48]. Additional nonmotor symptoms include REM sleep behavior disorder (RBD) and psychiatric manifestations, specifically major depression. Dementia is a key feature of *SNCA* triplication cases, and occurs early in the disease, with cognitive deficits consistent with dementia in PD [47,49,50]. Brain neuroimaging reveals frontoparietal atrophy [50].

Duplications of the *SNCA* locus are more common and are characterized by heterogeneous and generally milder clinical manifestations. Both familial and sporadic cases have been reported. In addition, unaffected carriers have been identified at advanced ages, suggesting reduced penetrance. The clinical phenotype is highly heterogeneous, with a benign form and typical late-onset motor involvement resembling sporadic PD on one end and a catastrophic course resembling *SNCA* gene triplication on the other [51,52,53,54]. Nonmotor symptoms such as depression, psychosis, and dysautonomia were present in the majority of the reported cases, while the presence of cognitive decline is variable with some patients presenting PD dementia. Kara and co-authors described a family with *SNCA* duplication presenting with parkinsonism and frontotemporal dementia (FTD) with severe anxiety and features of obsessive–compulsive disorder [55].

##### *PRKN* Mutation Carriers

Parkin protein is an E3 ubiquitin ligase and plays a role in mitophagy and the proteasome degradation pathway. It is encoded by the *PRKN* gene with an autosomal recessive form of inheritance. Mutations in the *PRKN* gene are the most common cause of early-onset PD, with a global distribution. The clinical phenotype of Parkin-PD is early-onset parkinsonism, often starting in the 30–40 s. Clinical features include a rather benign course and slow progression, limb dystonia, and a relative higher incidence of dyskinesias after L-Dopa therapy compared to the idiopathic form [56]. Both point and dosage pathogenic mutations have been reported. Homozygous and compound heterozygous mutations are considered to be pathogenic but the clinical importance of individual heterozygous mutations is still controversial [57].

PD harboring *PRKN* mutations is generally considered benign in terms of cognitive functions [2,3]. Studies assessing cognition in *PRKN* carriers are limited [2,3]. In a study using mainly MMSE or MoCA tests, the cognitive profile of *PRKN* carriers was rather close to that in iPD [12], but MMSE or MoCA are not sensitive enough to thoroughly evaluate mild cognitive differences. Using more elaborate neuropsychological assessment, two research groups [58,59] did not find marked differences in cognitive performance between PRKN-PD and iPD. Other studies, however, showed that *PRKN* carriers had superior scores on tests of visuospatial skills, attention, and memory compared to iPD patients [60]. Another single-center report also suggests that PRKN-PD has a rather benign cognitive profile. But, it should be taken into account that the study used only global cognitive function tests, and did not include a control iPD group [61]. The neuropathological findings observed in *PRKN*-PD, exhibiting neuronal loss in the substantia nigra, without LB pathology in most of the cases or with LBs restricted to brainstem regions, might explain the observed cognitive performance stability. It appears, therefore, that cognitive function in *PRKN*-PD is comparable or superior to that in iPD. Lack of consistent results across studies may be due to the fact that some studies have also included heterozygous carriers, in whom the link to *PRKN*-related pathogenicity is still elusive. A detailed neuropsychological evaluation in patients carrying biallelic pathogenic *PRKN* mutations, compared to age- and sex-matched iPD patients, is required to confirm a favorable outcome in cognitive functions in this group [62].

##### *PINK1* Mutation Carriers

Mutations in the *PINK1* gene are the second most common cause of autosomal recessive early-onset PD and account for 1–8% of familial early-onset PD. The clinical phenotype is similar to the disease in *PRKN* carriers and is characterized by early-onset parkinsonism, effective response to levodopa, slow disease progression, and occasional dystonia. Since PD with *PINK1* mutations is rare, data comparing cognitive function between *PINK1*-PD and iPD are scarce [2,3]. PINK1 PD cases are reported to have only MCI, and a systematic review of genetic autosomal recessive PD patients reported significant cognitive deficits in only 14% of *PINK1*-PD patients [56]. Notably, a large multicenter study reported cognitive decline in 25%, which is a percentage higher than that for *PRKN* mutation carriers [63]. Neuropathological data in *PINK1* mutation carriers with PD are also scarce, and based on the few cases reported, some have relatively restricted LB pathology [64]. Overall, the issue of whether cognitive deterioration is a significant nonmotor manifestation in *PINK1*-related PD is still elusive.

##### *DJ-1* Mutation Carriers

*DJ-1* is the third most common cause of autosomal recessive EOPD, with a mean age of onset of 27 years. To date, approximately 20 pathogenic variants have been isolated, and mutations are found in less than 1% of autosomal recessive PD cases. The motor phenotype of *DJ-1* is similar to that of *PRKN* and *PINK1* mutation carriers, including dystonia and L-Dopa-induced dyskinesias [56]. Neuropathological studies of *DJ-1* mutation carriers have revealed α-synuclein accumulation and LBs [65]. The phenotype is variable and includes cognitive decline, anxiety, depression, and even psychotic features [2,3,66]. Kasten and co-authors, in a systematic review of the MDS gene database that was assessing genetic and phenotypic data, have reported cognitive decline in 17% of *DJ-1* patients [56]. Due to the rarity of this mutation, it is still unclear whether cognitive dysfunction is a significant feature in *DJ-1* mutation carriers [56].

#### 3.1.3. Typical or Early-Onset Genetic PD with Variable Cognitive Decline

##### *GBA* Mutation Carriers

The *GBA* gene encodes the lysosomal enzyme glucocerebrosidase, which is involved in glucosylceramide metabolism. Pathogenic mutations in both *GBA* alleles cause the lysosomal storage disorder Gaucher disease, while heterozygous *GBA* mutations represent the most common genetic risk factor for PD and dementia with Lewy bodies (DLB). *GBA* mutations increase the risk of PD by about four- to five-fold [67]. The clinical phenotype of PD associated with PD mutations (*GBA*-PD), compared to iPD, is characterized by a younger age of onset, an akinetic–rigid disease subtype, and more severe nonmotor symptoms, including cognitive changes and occasionally psychotic features [68,69]. In most studies, the incidence of cognitive impairment or dementia is significantly higher in *GBA*-PD compared to iPD [70]. In a study in a Greek cohort, dementia was more common and MMSE scores lower in *GBA*-PD compared to iPD matched for age and disease duration [71]. Furthermore, in a prospective study, dementia and psychosis developed significantly earlier in the disease course in *GBA*-PD compared to iPD [72]. Only a few studies in *GBA*-PD used an extensive neuropsychological battery; in these, the *GBA*-PD group performed worse than non-carriers in different cognitive domains, including non-verbal memory, working memory, executive function, and visuospatial skills [73,74]. The particular *GBA* mutation appears to play a role in the extent of cognitive impairment in *GBA*-PD. The risk of dementia is modulated by the type of mutation in *GBA* mutation carriers, with a higher risk for dementia in individuals with severe (p.L444P, p.G377S, IVS10+1G > T) compared to mild mutations (N370S) [75]. Interestingly, genome-wide association studies (GWASs) including longitudinal data from multiple cohorts showed that even the p.E326K *GBA* variant, which is not associated with Gaucher disease, but only with an increased risk of PD, led to more frequent cognitive impairment compared to iPD [76]. Neuropathological studies of *GBA*-PD patients revealed widespread LB pathology, involving both the brainstem and the cortex, potentially explaining the more severe cognitive dysfunction [77]. AD pathology is rare in dementia associated with *GBA*-PD, and AD-related biomarkers are infrequent in the CSF of such patients [78]. Consistent with the lack of association of Aβ pathology with *GBA*-PD, *APOE* E4 status does not predict dementia in this group, in contrast to iPD [19].

In conclusion, *GBA*-PD is generally associated with more severe cognitive impairment compared to iPD, particularly in executive and visuospatial domains, and more rapid disease progression, while the severity of cognitive decline generally correlates with the severity of the particular mutation. Dementia mostly correlates with LBs, not Aβ or Tau pathology.

Table 1 summarizes the information about typical genetic forms of PD.

### 3.2. Genetic Background of Dementia with Lewy Bodies (DLB) and Its Effect on Cognitive Impairment

Dementia with Lewy bodies (DLB) is a progressive neurodegenerative disorder caused by the accumulation of alpha-synuclein in neuronal cells, leading to the disruption of extensive neuronal networks and the clinical expression of dementia at around the same time as parkinsonism (“one year rule”). After Alzheimer’s disease (AD), it is the second most prevalent neurodegenerative cause of dementia, accounting for about 4.2% of cases in community settings, and 7.5% within secondary care [79]. However, according to neuropathological studies, DLB might be underdiagnosed, and the more accurate percentage might be up to 20% [80]. The clinical diagnosis of DLB requires at least two of the following main symptoms: fluctuations in cognitive performance, visual hallucinations, parkinsonism, and rapid eye movement (REM) sleep behavior disorder (RBD) [81]. Although AD and DLB are two different diseases, a significant neuropathological overlap is often observed, including amyloid plaques and alpha-synuclein aggregation [82].

The clinical phenotype of DLB includes cognitive, behavioral, and motor symptoms. Cognitive impairment is characterized by fluctuations mainly in attention, executive function, and visuospatial and visuoconstructive abilities, and variable episodic memory deficits [83,84]. During the course of the disease, language is also affected in word and sentence comprehension, word production, spontaneous speech, and reading [83].

The most well-established genetic factors associated with DLB include the *SNCA*, *GBA*, and *APOE* genes [82,85]. The first GWAS of DLB confirmed its association with *APOE*, *SNCA*, and *GBA* gene variants [86].

#### 3.2.1. *APOE*

*APOE* E4 is the most common genetic susceptibility factor for AD. However, a growing body of evidence suggests its relationship with more severe cognitive impairment in various neurodegenerative diseases, including DLB, possibly because of its effects on amyloid plaque formation and tau-related pathology [87]. Some studies have shown that *APOE* E4 may increase the risk of DLB [21,88], possibly due to the shared clinical and pathological characteristics between AD and DLB, including the strong association between amyloid and Lewy body pathology [89]. However, a recent study questions whether *APOE* E4 constitutes a risk factor for DLB, and attributes the common co-occurrence to the common comorbidity of DLB with AD. According to that report, due to the underestimated comorbidity, *APOE* e4 might be—mistakenly—reported as a risk factor for DLB, while only increasing the risk of AD [90]. From another perspective, Dickson et al. [20] proposed that *APOE* E4 may alter the risk of DLB by its direct effect on LBs, in addition to AD, pathology. A study by Pilai et al. [84] found that the odds of amnestic and non-amnestic initial symptoms vary according to *APOE* E4 status and the underlying neuropathology. In particular, initial amnestic and/or visuospatial, but not language symptomatology, was associated with the presence of *APOE* E4 in pure AD neuropathology or with co-existing LB pathology. Further studies are needed to elucidate the role of *APOE* e4 4 as a risk factor for DLB and its relationship with the clinical phenotype, in the absence or presence of AD co-pathology [84,90].

#### 3.2.2. *SNCA*

α-synuclein, encoded by *SNCA*, plays a pathogenic role in DLB, contributing to the abnormal protein accumulation and the formation of LBs. As mentioned, pathogenic variants in *SNCA* are associated with a wide clinical heterogeneity and phenotypic spectrum including PD, DLB, PDD, a Multiple Systems Atrophy (MSA)-like phenotype, and FTD [25,33,43,91]. Protein aggregations in DLB can disrupt synaptic function and connectivity in the cortex and the limbic system, contributing to the fluctuating cognition, memory decline, and executive dysfunction [25,43]. Current research is focused on the interplay between alpha-synuclein, tau, and amyloid, which may synergistically exacerbate neurodegeneration in DLB [43,91]. GWASs also suggest [92] that DLB may have a shared genetic background with PD and AD, including a significant association with variations in the *SNCA* gene [93].

#### 3.2.3. *GBA*

*GBA* encodes an enzyme involved in lipid metabolism, and *GBA* mutations may lead to dysfunctional glucocerebrosidase enzyme activity. As a result, glucocerebroside accumulates in lysosomes, which contributes to neurodegeneration by disrupting cellular function [94]. The *GBA* gene has been widely explored as a genetic risk factor of synucleinopathies, including DLB, although the underlying mechanisms remain unclear. A recent meta-analysis by Liu et al. [95] confirmed that *GBA* may increase the risk of DLB. In addition, consistent with the findings in PD mentioned earlier, within the DLB group, *GBA* mutation carriers had an earlier onset of the disease, more severe cognitive impairment, and more rapid progression compared to the group of non-carriers [95]. Similarly, in another study by Walton et al. [77], *GBA* gene mutations were associated with more severe cognitive and motor impairment in DLB [96]. According to Walton et al. [77], *GBA* mutations were also associated with greater Lewy body and lower AD pathology. Collectively, these findings suggest that *GBA* mutations detrimentally affect the time of onset, severity, and progression of cognitive decline in DLB.

### 3.3. Genetic Forms of Atypical Parkinsonian Syndromes Associated with Cognitive Decline

#### 3.3.1. Corticobasal Syndrome

CBS is characterized by a combination of motor (extrapyramidal) and cognitive (cortical) symptoms and signs. The potential underlying pathologies vary, with corticobasal degeneration (CBD) and AD being present more commonly; TDP-43 pathology may also occasionally underlie CBS. Main clinical manifestations include asymmetric motor symptoms such as dystonia, rigidity, tremor, and the alien limb phenomenon, as well as cognitive and behavioral symptoms such as parietal cortex dysfunction, language impairment, and memory deficits [97]. Although overall a genetic etiology in CBS is infrequently encountered, genetic factors have been increasingly shown to play a critical role in its pathogenesis, including mutations in the progranulin gene (*GRN*) and the *MAPT* gene and expansions in the human chromosome 9 open reading frame 72 (*C9orf72*) locus. According to the systematic review by Arienti et al. [98], *GRN* was the commonest gene involved, with 28 cases (48%) out of 23 families, followed by *MAPT* (16%), *C9ORF72* (10%), and *PRNP* (7%). For the purposes of this review, GRN, MAPT, and *C9ORF72* will be discussed in the following sections.

*GRN*

Progranulin (PGRN, encoded by the *GRN* gene) plays an important role in the development, survival, function, and maintenance of neurons and microglia in the mammalian brain. PGRN contributes to the regulation of lysosomal biogenesis, neuroinflammation, stress responses, repair, and the aging process. Loss-of-function mutations in the *GRN* gene have been described as causes of FTD or neuronal ceroid lipofuscinosis, acting in a dosage-dependent manner [99].

*GRN* mutations are inherited in an autosomal dominant manner, and they have been described as a major cause of FTD [100] with ubiquitin and TDP-43-immunoreactive and tau-negative neuronal inclusions [101], along with a variety of other clinical phenotypes, including CBS [102,103].

In the review by Arienti et al. [98], in which *GRN* mutations occurred in about 48% of genetic CBS cases, a positive family history of a neurological disorder was mentioned in about two thirds. Approximately half of these familial cases indicated a phenotype similar to CBS, while in *GRN*-FTD this was reported in at least one family member in about one third of the cases [104]. Age of disease onset in *GNR* mutation carriers was 58 years, which does not deviate from that of sporadic CBS [98].

Regarding the clinical phenotype, patients with CBS and *GRN* mutations display visuospatial impairment, behavioral changes, aphasia, and/or language deficits. Because of the shared initial neuropsychological deficits, the clinical profile of GRN mutation carriers is often later differentiated to CBS or FTD [103,105]. What is more, the frequency of the characteristic symptoms and signs of CBS (cortical and extrapyramidal) does not seem to differ between *GRN* mutation carriers and non-carriers [98].

Concerning neuroimaging data, *GRN* carriers show asymmetrical cortical atrophy contralateral to the most impaired clinical side. The findings are mainly in the fronto–temporo–parietal cortex [106,107,108], which corroborates previous studies highlighting the association of *GRN* mutations with asymmetric and widespread atrophy in the temporal and parietal lobes [109,110].

*MAPT*

The *MAPT* gene encodes the tau protein, which contributes to the maintenance of the stability of microtubules in neurons. *MAPT* gene mutations may lead to tauopathies, in which tau becomes hyperphosphorylated and begins to form neurofibrillary tangles (NFTs), which play an important role in the neurodegenerative process [98].

Compared to non-carriers, CBS patients carrying *MAPT* gene mutations manifest the disease earlier, at about 48.2 years of age. *MAPT* mutations seem to be highly penetrant, as a family history of parkinsonism or dementia is frequently observed among *MAPT* mutation carriers [111].

Among the different genetic forms of CBS, carriers of *MAPT* mutations exhibit the highest prevalence of tremor, dystonia, and oculomotor dysfunction, highlighting the pervasive presence of motor deficits. Cognitive domains frequently impaired in *MAPT* carriers include memory, language, and cortical sensory functions [98].

Neuroimaging evidence suggests that *MAPT* mutation carriage is associated with bilateral frontal and/or parietal atrophy, and less frequently mild asymmetry in these areas, or the fronto–temporo–parietal regions [112]. These findings suggest that in some cases *MAPT* gene mutations may be linked to bilateral cortical atrophy, with prominent movement disorders, although further studies with larger sample sizes are needed to clarify the clinical–genetic correlations in *MAPT*-associated CBS.

*C9orf72*

The *C9orf72* genetic locus (chr9p21.2) encodes the *C9orf72* protein, which is expressed in the cytoplasm of neurons and presynaptic terminals. *C9orf72* protein is thought to play a key role in the regulation of endosomal trafficking [113], RNA processing [114], and nucleocytoplasmic transport [115]. A pathological hexanucleotide repeat expansion (GGGGCC), in the intronic region of the *C9orf72* locus—exceeding 30 repeats—is the most common genetic cause of familial autosomal dominant amyotrophic lateral sclerosis (ALS) as well as frontotemporal dementia (FTD) with ALS [116]. Notably, approximately 35% of patients with *C9orf72* mutations display parkinsonian symptoms [117].

According to Arienti et al. [98], the mean age of disease onset is 50.2 years, with a positive family history for dementia. Clinically, CBS patients carrying *C9orf72* mutations demonstrate more severe cognitive impairment, frequently presenting with a frontal dysexecutive syndrome, attention deficits, and behavioral symptoms such as apathy, disinhibition, mutism, ritualistic behaviors, and hyperphagia, compared to non-carriers. Neuroimaging data depict asymmetric frontotemporal atrophy on MRI, with generalized cortical atrophy being reported in two cases [98]. It is proposed that *C9orf72* carriers have widespread atrophy following an antero–posterior course in time, while the cerebellum also becomes affected [110].

#### 3.3.2. Progressive Supranuclear Palsy

PSP is a rare neurodegenerative disorder characterized by supranuclear gaze palsy, rigidity, and postural instability. PSP is associated with accumulation of tau protein in the basal ganglia, brainstem, and frontal cortex. Despite the variability in the clinical phenotypes associated with PSP, the most classic phenotype remains Richardson’s syndrome [118]. Recently, updated diagnostic criteria were formulated for a more accurate classification [119]. Although PSP is often a sporadic disease, there are occasionally encountered familial forms usually associated with *MAPT* and leucine-rich repeat kinase 2 (*LRKK2*) gene mutations.

*MAPT*

The prevalence of *MAPT* mutations in PSP varies between 0.6% and 14.3% [120,121,122,123]. Among the 15 different mutations of the *MAPT* gene in patients with PSP, the most common one is located at codon 279, reported in 11 cases [118]. Age of disease onset is approximately 44.8 years in PSP patients carrying *MAPT* mutations, which is significantly earlier compared to non-carriers [118]. There is often a family history of parkinsonism, dementia, or other neurodegenerative disorders [118]. Regarding the clinical phenotype, initial symptoms include both motor and cognitive deficits. In particular, as described in a review of Wen et al. [118], which included studies on both clinically diagnosed PSP and PSP-like syndromes, early symptoms may include parkinsonism, falls, dysarthria, micrographia, apathy, oscillopsia, gait instability, bradykinesia, and dystonia. Additionally, supranuclear gaze palsy, behavioral disturbances, and cognitive dysfunction are common. Hence, while the initial presentation is variable, it usually consists of parkinsonism, gait disturbances, and frontal executive dysfunction.

*LRKK2*

Leucine-rich repeat kinase 2 (*LRRK2*) is one of the most common genetic causes of Parkinson’s disease (PD), with over five pathogenic *LRRK2* mutations identified in both familial and sporadic PD cases [4,124]. While some studies have not detected *LRRK2* mutations in PSP, others have identified specific variants in rare cases, including R1441C, R1441H, G2019S, T2310M, and A1413T. Compared to non-carriers, the age of disease onset is older for patients with PSP carrying *LRRK2* gene mutations (mean: 72.3 years), with the predominant clinical phenotype being parkinsonism, frequently leading to an initial misdiagnosis of PD [118]. However, *LRRK2* remains a rare genetic contributor to PSP, and further studies are needed to clarify its role in disease pathogenesis and phenotypic expression.

### 3.4. Genetic Forms of Frontotemporal Dementia with Parkinsonism

FTD is a clinically, pathologically, and genetically heterogeneous group of neurodegenerative diseases, characterized by progressive deficits in cognition and behavior. About 40% of patients with FTD are familial cases, reporting a family history of a cognitive and/or movement disorder [125]. Most patients with FTD display parkinsonian features during the course of the disease, while they may be also evident at initial stages, leading to diagnostic challenges [126]. Bradykinesia, rigidity, and postural instability are occasionally present in FTD, particularly in the behavioral variant of FTD (bvFTD) and progressive non-fluent/agrammatic aphasia (PNFA) [126]. The phenomenology of parkinsonism in FTD usually differs from that of Parkinson’s disease (PD), while it resembles atypical parkinsonian syndromes including Richardson’s syndrome and CBS [126]. In particular, parkinsonism in FTD is more commonly axial and symmetric, with poor response to dopaminergic therapy, while the presence of tremor is less common [126].

The clinical presentation of patients with FTD may provide clues to specific gene mutations, which can also reflect the underlying neuroanatomical and pathophysiological deficits and provide insights into potential therapeutic approaches [125]. Approximately 50% of the heritable cases are caused by mutations in three genes (*MAPT*, *PGRN*, and *C9orf72*) [125], which often present with a combination of cognitive and behavioral deficits accompanied to a variable degree by parkinsonian features.

#### 3.4.1. *MAPT* Gene Mutations

Mutations in the microtubule-associated protein tau (*MAPT*) gene, located on chromosome 17, are a well-established cause of FTD with parkinsonism [127]. Several different mutations have been detected in this gene, which may impair the function of the protein or alternative splicing, resulting in abnormal tau aggregation in neurons and glial cells [127]. *MAPT* mutations are often associated with differences in the 3R/4R isoform ratio, the structure of tau filaments, and sometimes the clinical endophenotype [127].

*MAPT*-associated FTD cases are characterized by clinical heterogeneity, and they may resemble sporadic FTD [128]. According to a recent systematic review, the most common phenotype is bvFTD, followed by PNFA and the semantic variant of Primary Progressive Aphasia (svPPA), while one in five patients presented with a phenotype on the CBS-PSP spectrum [128]. Parkinsonism in *MAPT*-associated FTD cases may precede the behavioral and cognitive symptoms even by several years or come later during the disease course [129]. Several mutations in *MAPT*, particularly in exon 10, have been associated with parkinsonian features. Although the symmetric akinetic–rigid parkinsonian syndrome is the commonest manifestation, with varying degrees of severity, the clinical features may differ in specific *MAPT* mutations [129]. Richardson’s syndrome related to *MAPT* mutations is usually incomplete or atypical, accompanied by the cognitive and behavioral characteristics of bvFTD and possibly a positive family history, while CBS occurs less frequently [129].

#### 3.4.2. *GRN* Gene Mutations

Mutations in the gene encoding progranulin (*GRN*), which is also located on chromosome 17 and close to the *MAPT* gene, exist in 5–10% of individuals with FTD and in approximately 20% of familial cases [126]. Loss-of-function mutations in the *GRN* gene result in lower levels of progranulin, which plays a key role in lysosomal function and neuronal survival [129]. Progranulin is also implicated in the cleavage of the ubiquitin-binding protein TAR DNA-binding protein 43 (TDP-43) [129]. Unlike FTD cases related to *MAPT* that are characterized by tau pathology, TDP-43 proteinopathy underlies PGRN-associated FTD.

Compared to *MAPT*, *GRN* mutations are characterized by higher phenotypic variability, including FTD, amyotrophic lateral sclerosis (ALS), typical Parkinson’s disease (PD), and dementia with Lewy bodies (DLB) [103]. Hallucinations are more common in *GRN*-FTD cases compared to other genetic forms of FTD, often resembling DLB, and it has been proposed that they could be a clinical characteristic discriminating PGRN from other genetic forms of FTD [129]. Patients with *GRN* mutations often display asymmetric brain atrophy and parieto–occipital involvement, resulting in asymmetric visuospatial impairment and hemi-neglect, limb apraxia, and potentially CBS [129]. Patients with the most common *GRN* mutation, Arg493X, more commonly present with a phenotype of bvFTD, followed by PNFA and CBS [126]. Postural or resting tremor, dystonia, and asymmetric parkinsonism might be also present [126,130]. In particular, CBS has been demonstrated as the most common presentation in patients carrying the Leu271LeufsX10 deletion in the *GRN* gene [103]. In contrast to MAPT, Richardson syndrome is rarely seen in cases with *GRN* mutations [129].

#### 3.4.3. *C9orf72* Genetic Locus Mutations

*C9orf72* hexanucleotide (GGGGCC) repeat expansions (>30 repeats) are the most common cause of FTD associated with ALS [131]. This hexanucleotide repeat expansion belongs to a non-coding region of the gene, and a combination of loss- and gain-of-function processes has been proposed as the underlying pathophysiological mechanism [131]. Neuropathologically, *C9orf72*-associated neurodegeneration invariably demonstrates TDP-43-positive aggregates; aggregation of dipeptide-repeat proteins (DPRs) is also seen, but, unlike TDP-43 aggregation, does not associate with regions of neurodegeneration [132].

There is prominent clinical heterogeneity among the *C9orf72* repeat expansion carriers, which may also be observed even within the same family [131]. Psychiatric manifestations are common, often accompanied by upper and lower motor neuron disease symptoms [131]. Parkinsonian features occur in about one third of cases with *C9orf72* mutations [129], and they can be asymmetric or symmetrical, with bradykinesia, rigidity, and postural instability and less commonly with postural, action, and, rarely, resting tremor [126]. *C9orf72* has been linked to typical PD, DLB, Richardson’s syndrome, and CBS [131]. Notably, autonomic dysfunction and ataxia might accompany the *C9orf72*-related parkinsonism, resembling MSA [133].

#### 3.4.4. Other Mutations Related to FTD and Parkinsonism

Mutations in the *CHMP2B* gene, which is located on chromosome 3 and encodes the charged multivesicular body protein 2b, have been linked with FTD accompanied by parkinsonism occurring later during the course of the disease [129]. Common movement disorders in patients with *CHMP2B* mutations include myoclonus, dystonia, and stereotypical behavior, while Richardson’s syndrome and CBS represent rare manifestations [129].

The Fused-in-Sarcoma (*FUS*) gene, encoding the RNA-binding protein FUS, located on chromosome 16, is also implicated in FTD, and it is infrequently accompanied by parkinsonism [129]. Postural tremor can be present in patients with *FUS* mutations, which may resemble essential tremor [129].

Valosin-containing protein (*VCP*) gene mutations, located on chromosome 9, have been linked to a multisystem disorder characterized by early Paget disease of the bone, inclusion body myopathy (IBM), and FTD [134]. These mutations have also been associated with parkinsonian features and motor neuron disease. Notably, the R191Q *VCP* mutation has been specifically related to parkinsonism, which might be the only initial symptom [134].

Mutations in the *TARDBP* gene, located on chromosome 1 and encoding the TDP-43 protein, have been primarily linked to FTD-ALS [129]. There are also several reports connecting *TARDBP* gene mutations, especially of A382T87, A315E, and N267S, with FTD and related parkinsonism [129].

TREM-2 is a protein involved in microglial function, while its dysregulation is considered to result in impaired phagocytosis and deposition of necrotic debris [135]. TREM-2 is encoded by the *TREM2* gene on chromosome 6, and *TREM2* mutations have been linked to FTD with parkinsonism, sometimes accompanied by myoclonus and alien limb syndrome [129].

Mutations in the TANK-binding kinase 1 (*TBK1*) gene are mainly related to FTD-ALS, but they have also been associated with parkinsonian features, including bradykinesia, rigidity, and tremor as well as a CBS-PNFA syndrome [126].

Table 2 summarizes the information about atypical genetic forms of PD.

## 4. Conclusions

Genetic background is likely to be able to explain some of the heterogeneity in degree of intellectual impairment among patients with PD or atypical parkinsonism.

Studies focusing on the genetics of PD can be categorized as either focused on carriers of known mutations or focused on more common variants in iPD. In monogenic forms, specific mutations correspond to a defined pattern of cognitive impairment. Specifically, dementia is rare in PD with *PRKN* mutations, and less common in PD with *LRRK2* mutations than in the idiopathic form. It is probably more common in carriers of *GBA* or *SNCA* mutations. The stronger the association between genotype and α-synuclein pathology, the greater the risk for cognitive decline.

As our knowledge of polygenic risk in PD and atypical parkinsonian syndrome expands, we will be able to better understand additional effects of genetic background on the development of cognitive disorder or dementia in such patients.

Future studies should focus on bridging the gap between clinical phenotypes and genetic background, in order to improve and personalize the diagnostic work-up and contribute to the success of relative clinical trials. Towards this direction, the use of advanced genetic sequencing technologies should be expanded, in order to detect novel genetic mutations, understand their functional implications, and establish their association with the clinical presentation. The integration of genetic evidence, advanced neuroimaging data—including structural and functional MRI or PET neuroimaging—and fluid biomarkers—including amyloid beta, phosphorylated tau, a-synuclein, neurofilament light chain, and the Seeding amplification a-synuclein assay (SAA)—would also enable the elucidation of the underlying neurobiological and neuroanatomical mechanisms across the different parkinsonian syndromes. In addition, through the literature review it became evident that studies focusing on the cognitive correlations with genetic aspects tend to focus on global screening tools such as MMSE and MoCA, rather than thorough neuropsychological assessments. It would be important for future studies to include more thorough neuropsychological evaluations in order to have a more detailed picture of the clinical phenotype of each patient. This would better differentiate between cognitive profiles and could be clinically relevant when considering differential diagnostics in atypical PD. Further studies exploring the functional consequences of known genetic mutations, including those in *SNCA*, *GBA*, and *MAPT*, would enhance our understanding of their pathogenic implication in lysosomal impairment, mitochondrial dysregulation, synaptic dysfunction, and neuronal loss leading to neurodegeneration. Moreover, longitudinal studies in patients with genetic forms of parkinsonism and cognitive decline would help to monitor the disease progression and document the trajectory of the progression of cognitive and movement deficits.

Given the strong clinical variability of most of the genetic mutations described above, future studies could also examine the potential environmental factors and epigenetic mechanisms that might contribute to this heterogeneity. In this context, whole genome methylation analysis in individuals with dementia has shown that the H1 *MAPT* haplotype as a risk factor for tauopathy may depend on methylation changes in the genetic locus encoding tau protein, suggesting that epigenetic modifications might affect genetic predisposition to PSP [136]).

Understanding the genetic causes of cognitive impairment associated with parkinsonism may also significantly aid in the development of novel personalized therapeutic strategies. In this regard, strategies such as antisense oligonucleotides (ASOs) against *C9orf72* repeat expansions or gene therapy to restore PGRN levels that have demonstrated promising results in preclinical studies, but are still lacking in evidence in the clinic, might eventually bear fruit [137,138]. Furthermore, immunotherapies such as tau monoclonal antibodies that are currently being explored might be proven effective in *MAPT*-related FTD with parkinsonian features. Besides the clinical implications related to earlier diagnosis, prognostication, and treatment, the early detection of genetic causes could enable the participation of patients in clinical trials.

## Figures and Tables

**Table 1:** Features of pathological mutations in the genes associated with PD and correlation with the degree of decline.

PD-Related Pathogenic Mutation	Impact on Cognitive Functions
*SNCA*—Point mutations	Heterogeneous phenotype depends on the mutation. Executive and visuospatial
	functions are more affected than
	episodic verbal memory.
*SNCA*—Duplication	Wide spectrum of manifestations from normal cognitive function to dementia in PD.
*SNCA*—Triplication	Early-onset dementia with cognitive deficits compatible with dementia in PD.
*LRRK2*	Relative preservation of cognitive functions compared to iPD.
*VPS53*	Signs of MCI, while some patients have normal functions.
*PRKN* (Parkin)	Cognitive functions are often normal even several years after the onset of PD.
*PINK1*	A subgroup of carriers presents cognitive impairment.
*DJ-1*	Some patients are without severe cognitive impairment. Other cases exhibit dementia.
*GBA*	In most patients, a greater rate of cognitive decline and faster progression is observed in patients with GBA mutations. Particularly pronounced deficits in visuospatial cognitive functions. Also, a risk factor for DLB.

**Table 2:** Pathological mutations in the genes that are associated with atypical parkinsonism.

Atypical Parkinsonism-Related Pathogenic Mutation	Motor–Cognitive Phenotype
*MAPT*	Apart from the typical FTD phenotype, one in five patients presented with a phenotype on the CBS-PSP spectrum. Parkinsonism in MAPT-associated FTD may precede the behavioral and cognitive symptoms and is often symmetric. Richardson’s syndrome related to MAPT mutations is usually incomplete or atypical (ranging from 0.6% to 14.3% of PSP cases), while CBS occurs less frequently.
*GRN*	High phenotypic variability, including FTD, ALS, DLB-like with hallucinations, or PD-like parkinsonism. CBS has been reported as a rather common presentation in patients carrying GRN mutations, often with marked language deficits, asymmetric visuospatial impairment, hemi-neglect, and limb apraxia. Richardson syndrome is rarely seen in GRN cases.
*C9ORF72*	The most common cause of FTD is associated with ALS. Parkinsonian features occur in about one third of cases. C9orf72 has also been linked to phenotypes of typical PD, DLB, Richardson’s syndrome, and CBS. Psychiatric manifestations are also common.
*LRRK2*	Rarely linked to PSP.
*CHMP2B*	FTD is accompanied by parkinsonism later during the disease, while Richardson’s syndrome and CBS represent rarer manifestations.
*FUS*	Implicated in FTD, also infrequently accompanied by parkinsonism (often with tremor).
*VCP*	Some mutations are linked to FTD with parkinsonian features and motor neuron disease.
*TARDBP*	Except for FTD-ALS, certain mutations are associated with FTD and related parkinsonism.
*TREM2*	Linked to FTD with parkinsonism.
*TBK1*	Apart from FTD-ALS, also associated with parkinsonian features as well as CBS-PNFA.

## Data Availability

There were no new data collected.

## Abbreviations

The following abbreviations are used in this manuscript:

AD	Alzheimer’s disease
ALS	amyotrophic lateral sclerosis
APOE	apolipoprotein E
bvFTD	behavioral variant of Frontotemporal Dementia
CBS	Corticobasal Syndrome
CBD	corticobasal degeneration
C9orf72	chromosome 9 open reading frame 72
DLB	Dementia with Lewy Bodies
DJ-1	PARK7 (related to Parkinson’s disease)
DPRs	dipeptide-repeat proteins
FTD	Frontotemporal Dementia
FUS	Fused-in-Sarcoma
GWAS	Genome-Wide Association Study
iPD	idiopathic PD (henceforth iPD)
LB	Lewy body
LRRK2	leucine-rich repeat kinase 2
MAPT	microtubule-associated protein tau
MCI	Mild Cognitive Impairment
MMSE	Mini Mental State Examination
MoCA	Montreal Cognitive Assessment
MSA	Multiple Systems Atrophy
PPMI	Parkinson’s Progression Markers Initiative
PNFA	progressive non-fluent/agrammatic aphasia
PD	Parkinson’s disease
PINK1	PTEN-induced kinase 1
PSP	Progressive Supranuclear Palsy
RBD	REM Sleep Behavior Disorder
SNCA	α-synuclein
svPPA	semantic variant of Primary Progressive Aphasia
TBK1	TANK-binding kinase 1
VCP	Valosin-containing protein
VPS35	vesicular sorting protein 35
AD	Alzheimer’s disease
ALS	amyotrophic lateral sclerosis
APOE	apolipoprotein E
bvFTD	behavioral variant of Frontotemporal Dementia
CBS	Corticobasal Syndrome
CBD	corticobasal degeneration
